# Impact of dose-dense neoadjuvant chemotherapy on pathologic response and survival for HER2-positive breast cancer patients who receive trastuzumab

**DOI:** 10.1038/s41523-021-00284-y

**Published:** 2021-06-11

**Authors:** Lize Wang, Yang Zhang, Yingjian He, Jinfeng Li, Tianfeng Wang, Yuntao Xie, Zhaoqing Fan, Tao Ouyang

**Affiliations:** Key Laboratory of Carcinogenesis and Translational Research (Ministry of Education/Beijing), Breast Cancer Center, Peking University Cancer Hospital & Institute, Beijing, China

**Keywords:** Chemotherapy, Breast cancer

## Abstract

To compare outcomes in patients with human epidermal growth factor receptor-2 (HER2)-positive breast cancer who received either dose-dense neoadjuvant chemotherapy (NAC) with trastuzumab or standard-interval chemotherapy with trastuzumab. Patients with HER2-positive breast cancer who received NAC, including epirubicin and cyclophosphamide followed by paclitaxel with trastuzumab were included. Patients were divided into either the dose-dense or standard-interval group. We compared pathologic complete remission (pCR), distant disease-free survival (DDFS), event-free survival (EFS), and breast cancer-specific survival (BCSS) between the two groups. Two hundred (49.6%) patients received dose-dense NAC, and 203 (50.4%) received standard-interval NAC. The pCR rate was 38.4% in the dose-dense group and 29.2% in the standard-interval group (*P* = 0.052). In patients with lymph node (LN) metastases, the LN pCR rate was 70.9% in the dose-dense group and 56.5% in the standard-interval group (*P* = 0.037). After a median follow-up of 54.6 months, dose-dense chemotherapy presented an improvement on DDFS (hazard ratio [HR] = 0.49, 95% confidence interval [CI]: 0.19–1.28, EFS (HR = 0.54, 95% CI: 0.24–1.21), and BCSS (HR = 0.41, 95% CI: 0.11–1.51), but the difference was not significant. Compared with standard-interval chemotherapy, dose-dense chemotherapy resulted in a superior 5-year DDFS (100% vs. 75.3%, *P* = 0.017) and 5-year EFS (96.9% vs. 78.3%, *P* = 0.022) in patients younger than 40 years. HER2-positive patients can achieve a higher LN pCR rate with dose-dense NAC than with standard-interval NAC with trastuzumab. Better survival may also be achieved with dose-dense chemotherapy with trastuzumab than with standard-interval chemotherapy with trastuzumab among young patients (age ≤ 40 years).

## Introduction

The Norton-Simon theory suggests that shortening the chemotherapy intervals but keeping the same dosage per cycle may increase the killing effect on tumor cells and reduce the regeneration between cycles^1,2^. The INT C9741 trial showed significant improvement in survival outcomes in the dose-dense treatment group compared with the standard-interval treatment group, which supported the Norton-Simon theory^3^. A previous meta-analysis from the Early Breast Cancer Trialists’ Collaborative Group suggested that dose-dense chemotherapy improved 10-year disease-free survival (DFS) and overall survival (OS) independent of the molecular type of breast cancer^4^. The above studies support the superior efficiency of dose-dense chemotherapy over standard-interval chemotherapy, and anti-HER2 therapy that has not been used for routine administration in most of the previous studies^3,5–10^. At present, trastuzumab is used as the standard adjuvant or neoadjuvant therapy for HER2-positive breast cancer^11–13^. However, no prospective study has explored the differences in outcomes between dose-dense and standard-interval regimens with the administration of trastuzumab. According to the retrospective results of the PANTHER trial and the GIM2 trial, it remains controversial whether dose-dense chemotherapy leads to superior survival compared with standard-interval chemotherapy with trastuzumab in HER2-positive breast cancer^14,15^.

The purpose of this study was to investigate the differences in pathologic response and survival outcomes between dose-dense and standard-interval chemotherapy in patients with HER2-positive breast cancer who receive neoadjuvant chemotherapy (NAC), including an anthracycline-based regimen followed by paclitaxel and trastuzumab.

## Results

### Patient characteristics

**Fig. 1:**
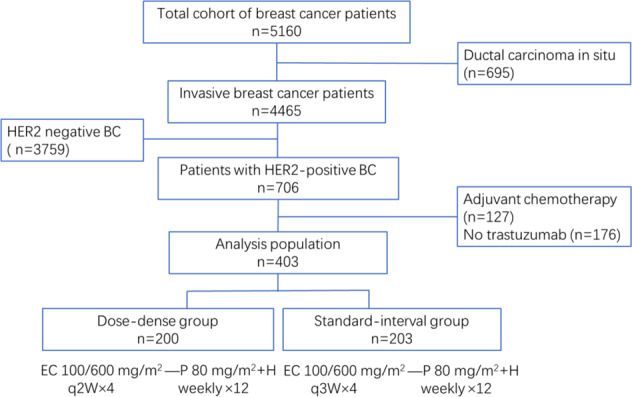
Flowchart of patient selection in this study. On the basis of the eligibility criteria, a total of 403 patients meet the criteria for inclusion and are divided into the dose-dense group and the standard-interval group. BC breast cancer, HER2 human epidermal growth factor receptor-2, E epirubicin, C cyclophosphamide, P paclitaxel, H trastuzumab.

Between March 2012 and December 2016, 5160 patients with primary breast cancer were diagnosed at our institution. Patients who did not meet the inclusion criteria were excluded, and 403 consecutive cases were analyzed (Fig. 1). All 403 patients received NAC and trastuzumab anti-HER2 therapy. Of the 403 eligible patients, 200 (49.6%) received dose-dense neoadjuvant treatment (dose-dense group), and 203 (50.4%) received standard-interval neoadjuvant treatment (standard-interval group). All patients were female, with a median age of 51 (range, 23–70) years. In the entire cohort, 75 (18.6%) patients were aged ≤ 40 years. These young patients accounted for 16% of the dose-dense group and 21.2% of the standard-interval group. In the dose-dense group, 119 (59.5%) patients were node-positive versus 115 (56.7%) in the standard-interval group (Table 1).

**Table 1:** Baseline clinicopathologic characteristics of HER2-positive breast cancer patients with exposure to trastuzumab.

Characteristic	Dose-dense group (*n* = 200) *N* (%)	Standard-interval group (*n* = 203) *N* (%)	*P-*value*
Age at diagnosis			0.181
≤ 40 years	32 (16.0)	43 (21.2)	
> 40 years	168 (84.0)	160 (78.8)	
Tumor size			0.178
T1	40 (20.0)	57 (28.1)	
T2	136 (68.0)	127 (62.6)	
T3−4	21 (10.5)	18 (8.9)	
Unknown	3 (1.5)	1 (0.5)	
Lymph nodes status			0.560
Negative	80 (40.0)	87 (42.9)	
Positive	119 (59.5)	115 (56.7)	
Unknown	1 (0.5)	1 (0.5)	
Hormone receptor status^a^			0.985
Negative	79 (39.5)	80 (39.4)	
Positive	121 (60.5)	123 (60.6)	
Tumor grade			0.464
G1−2	141 (70.5)	151 (74.3)	
G3	51 (25.5)	46 (22.7)	
Unknown	8 (4.0)	6 (3.0)	
Type of surgery			0.341
BCT	71 (35.5)	63 (31.0)	
Mastectomy	129 (64.5)	140 (69.0)	
Adjuvant endocrine therapy			0.177
Tamoxifen	80 (66.1)	71 (57.7)	
Aromatase inhibitor	41 (33.9)	52 (42.3)	
Ovarian function suppression			0.056
Yes	2 (1.7)	8 (6.5)	
No	119 (98.3)	115 (93.5)	

*BCT* breast-conserving therapy.

*P-value < 0.05 is statistically significant.

^a^Estrogen receptor and/or progesterone receptor status.

Comparison of the baseline characteristics between the two groups showed no significant differences in age at diagnosis, tumor size, histological grade, hormone receptor status, lymph node (LN) status, and type of surgery. The dose intensity of epirubicin in the dose-dense group was 1.5 times that in the standard-interval group. The mean number of cycles of epirubicin administration was 3.98 in the dose-dense group and 3.67 in the standard-interval group. The mean number of cycles of paclitaxel administration was 4.01 in the dose-dense group and 4.02 in the standard-interval group. The rate of adjuvant ovarian function suppression delivered in patients aged ≤40 years was 4% in the dose-dense group and 17.9% in the standard-interval group (*P* = 0.173).

### Pathologic complete remission

For the 400 cases available with postoperative pathologic evaluation, the pathologic complete remission (pCR) rate (ypT0) was 38.4% in the dose-dense group and 29.2% in the standard-interval group, which was not significant (*P* = 0.052). LN metastases were confirmed by sentinel LN biopsy (SLNB) for 39 patients whose post-NAC pathologic evaluations were unavailable. For the 195 cases with LN metastases, the LN pCR rate (ypN0) was 70.9% in the dose-dense and 56.5% in standard-interval groups, which was a significant difference (*P* = 0.037). The total pCR rate was 49.2% in the dose-dense group and 47.2% in the standard-interval group, which was not a significant difference (*P* = 0.707). Hormone-receptor-negative patients had higher pCR rates compared with hormone-receptor-positive patients (Table 2). The pCR rate was similar in the dose-dense group and the standard-interval group, stratified by hormone receptor status (Table 3). The tumor cells of three patients were found by core needle biopsy only in axillary LNs, and the information on the pathologic remission of the breast tumor was unavailable.

**Table 2:** Pathologic complete responses by subgroups.

Pathologic remission	Subgroup by chemotherapy			Subgroup by HR status			Subgroup by LN status		
	Dose-dense *N* (%)	Standard-interval *N* (%)	*P-*value*	HR-positive *N* (%)	HR-negative *N* (%)	*P-*value*	LN-positive *N* (%)	LN-negative *N* (%)	*P-*value*
ypT0			0.052			<0.001			0.115
Yes	76 (38.4)	59 (29.2)		62 (25.6)	73 (46.2)		71 (30.7)	64 (38.3)	
No	122 (61.6)	143 (70.8)		180 (74.4)	85 (53.8)		160 (69.3)	103 (61.7)	
ypT0/is			0.762			<0.001			0.006
Yes	102 (51.5)	101 (50.0)		99 (40.9)	104 (65.8)		103 (44.6)	98 (58.7)	
No	96 (48.5)	101 (50.0)		143 (59.1)	54 (34.2)		128 (55.4)	69 (41.3)	
ypN0			0.037			0.003			–
Yes	73 (70.9)	52 (56.5)		73 (57.0)	62 (77.5)		–	–	
No	30 (29.1)	40 (43.5)		55 (43.0)	18 (22.5)		–	–	
Total pCR			0.707			<0.001			<0.001
Yes	89 (49.2)	84 (47.2)		85 (38.8)	88 (62.9)		75 (39.1)	98 (58.7)	
No	92 (50.8)	94 (52.8)		134 (61.2)	52 (37.1)		117 (60.9)	69 (41.3)	

ypT0: no residual invasive or noninvasive tumor cells in the breast, ypT0/is: no residual invasive tumor cells in the breast, ypN0: no residual tumor cell in lymph nodes, total pCR: ypT0/is and ypN0.

*HR* hormone receptor, *LN* lymph node.

*P-value < 0.05 is statistically significant.

**Table 3:** Pathologic complete responses by chemotherapy, stratified by hormone receptor status.

Pathologic remission	Hormone receptor-positive			Hormone receptor-negative		
	Dose-dense group *N* (%)	Standard-interval group *N* (%)	*P-*value*	Dose-dense group *N* (%)	Standard-interval group *N* (%)	*P-*value*
ypT0			0.618			0.909
Yes	51 (42.5)	48 (39.3)		51 (65.4)	53 (66.2)	
No	69 (57.5)	74 (60.7)		27 (34.6)	27 (33.8)	
ypT0/is			0.210			0.113
Yes	35 (29.2)	27 (22.1)		41 (52.6)	32 (40.0)	
No	85 (70.8)	95 (77.9)		37 (47.4)	48 (60.0)	
ypN0			0.191			0.108
Yes	43 (62.3)	30 (50.8)		34 (85.0)	18 (70.0)	
No	26 (37.7)	29 (49.2)		6 (15.0)	12 (30.0)	
Total pCR			0.717			0.897
Yes	44 (40.0)	41 (37.6)		45 (63.4)	43 (62.3)	
No	66 (60.0)	68 (62.4)		26 (36.6)	26 (37.7)	

ypT0: no residual invasive or noninvasive tumor cells in the breast, ypT0/is: no residual invasive tumor cells in the breast, ypN0: no residual tumor cell in lymph nodes, total pCR: ypT0/is and ypN0.

*P-value < 0.05 is statistically significant.

### Survival outcomes

The median follow-up time in the entire cohort was 54.6 (range, 4–93) months. The median follow-up time was 49.7 (range, 12–91) months in the dose-dense group and 62.6 (range, 4–93) months in the standard-interval group. Ten (2.4%) patients were lost to follow-up. During follow-up, 20 patients underwent distant disease-free survival (DDFS) events, including six in the dose-dense group and 14 in the standard-interval group. Twelve patients experienced breast cancer-specific survival (BCSS) events, including three in the dose-dense group and nine in the standard-interval group. Twenty-eight event-free survival (EFS) events occurred: ten in the dose-dense group and 18 in the standard-interval group.

For all 403 patients, the 5-year DDFS was 96% (95% confidence interval [CI]: 92.7–99.3%) in the dose-dense group and 93.1% (95% CI: 89.4–96.8%) in the standard-interval group (*P* = 0.139). The 5-year EFS was 93.1% (95% CI: 88.8–97.4%) in the dose-dense group and 91.1% (95% CI: 87.0–95.2%) in the standard-interval group (*P* = 0.232). The 5-year BCSS was 98.3% (95% CI: 96.4–100.0%) in the dose-dense group and 95.7% (95% CI: 92.6–98.8%) in the standard-interval group (*P* = 0.191). The Kaplan–Meier survival analyses showed no significant differences in EFS, DDFS, or BCSS between the two groups. The comparisons of survival outcomes are shown in Table 4. The variables of age at diagnosis, tumor size, histological grade, hormone receptor status, LN status, type of chemotherapy, and type of surgery were included in the Cox multivariate analyses. Dose-dense chemotherapy presented an improvement on DDFS, hazard ratio [HR] = 0.49, 95% confidence interval [CI]: 0.19–1.28, *P* = 0.144), but which was not significant. A similar effect was observed for EFS and BCSS. The results also showed that age at diagnosis (>40 years vs. ≤40 years, HR = 0.36, 95% CI: 0.15–0.89, *P* = 0.027) and LN status (LN+ vs. LN−, HR = 3.11, 95% CI: 1.04–9.32, *P* = 0.043) were the factors influencing DDFS. Age at diagnosis (HR = 0.35, 95% CI: 0.16–0.74, *P* = 0.007) and LN status (HR = 4.59, 95% CI: 1.58–13.28, *P* = 0.005) were the factors influencing EFS. Age at diagnosis (HR = 0.35, 95% CI: 0.11–1.09, *P* = 0.070) and LN status (HR = 8.71, 95% CI: 1.12–67.54, *P* = 0.038) were the factors influencing BCSS. The Cox multivariate analyses are shown in Table 5.

**Table 4:** Comparisons of survival outcomes between the dose-dense group and the standard-interval group in the entire cohort and in positive nodes cohort, then stratified by age subgroups.

	Dose-dense group	Standard-interval group	*P-*value*
	5-year (95% CI)	5-year (95% CI)	
Entire cohort			
DDFS	96.0% (92.7−99.3%)	93.1% (89.4−96.8%)	0.139
EFS	93.1% (88.8−97.4%)	91.1% (87.0−95.2%)	0.232
BCSS	98.3% (96.4−>99.9%)	95.7% (92.6−98.8%)	0.191
Positive node cohort			
DDFS	94.4% (89.3−99.5%)	89.5% (83.7−95.3%)	0.170
EFS	89.5% (82.7−96.3%)	86.6% (79.4−92.6%)	0.258
BCSS	97.2% (94.1−> 99.9%)	93.5% (88.5−98.5%)	0.226
≤40 years cohort			
DDFS	100.0%	81.7% (69.2−94.2%)	0.017
EFS	96.9% (90.9−>99.9%)	78.3% (55.8−90.8%)	0.022
BCSS	100.0%	90.4% (80.1−>99.9%)	0.086
>40 years cohort			
DDFS	93.1% (87.1−99.1%)	92.9% (87.5−98.3%)	0.898
EFS	92.4% (87.4−97.4%)	94.6% (90.9−98.3%)	0.637
BCSS	98.0% (95.7−>99.9%)	97.0% (94.1−99.9%)	0.806

*CI* confidence interval, *DDFS* distant disease-free survival, *EFS* event-free survival, *BCSS* breast cancer-specific survival.

* *P*-value < 0.05 is statistically significant.

**Table 5:** Multivariate analyses for the effect of clinicopathologic characteristics and neoadjuvant treatment regimen on survival in the entire cohort.

Characteristic	DDFS		EFS		BCSS	
	HR (95% CI)	*P-*value*	HR (95% CI)	*P*-value*	HR (95% CI)	*P-*value*
Age at diagnosis		0.027		0.007		0.070
≤40 years	1		1		1	
>40 years	0.36 (0.15−0.89)		0.35 (0.16−0.74)		0.35 (0.11−1.09)	
Tumor size		0.486		0.210		0.453
T1	0.44 (0.09−2.08)	0.298	0.39 (0.11−1.46)	0.162	0.56 (0.08−3.90)	0.560
T2	0.53 (0.17−1.65)	0.273	0.45 (0.17−1.15)	0.094	0.41 (0.10−1.66)	0.211
T3−4	1		1		1	
Lymph nodes		0.043		0.005		0.038
Negative	1		1		1	
Positive	3.11 (1.04−9.32)		4.59 (1.58−13.28)		8.71 (1.12−67.54)	
Hormone receptor status		0.275		0.301		0.974
Negative	1		1		1	
Positive	1.76 (0.64−4.86)		1.58 (0.66−3.76)		0.98 (0.29−3.33)	
Tumor grade		0.923		0.761		0.647
G1-2	1		1		1	
G3	0.95 (0.31−2.91)		0.86 (0.32−2.32)		0.70 (0.15−3.22)	
Type of surgery		0.662		0.423		0.318
BCT	1		1		1	
Mastectomy	1.27 (0.44−3.64)		1.47 (0.57−3.78)		2.19 (0.47−10.20)	
Type of chemotherapy		0.144		0.133		0.180
Standard-interval	1		1		1	
Dose-dense	0.49 (0.19−1.28)		0.54 (0.24−1.21)		0.41 (0.11−1.51)	

*HR* hazard ratio, *BCT* breast-conserving therapy, *CI* confidence interval, *DDFS* distant disease-free survival, *EFS* event-free survival, *BCSS* breast cancer-specific survival.

* The excluding criteria for variables in the Cox proportional hazards model: *P*-value > 0.2.

### Subgroup analysis

Based on the multivariate analysis, age at diagnosis and LN status were the factors influencing survival. The subgroup analyses showed that dose-dense chemotherapy, rather than standard-interval chemotherapy, resulted in superior DDFS (5-year DDFS: 100% vs. 75.3%, *P* = 0.017) and EFS (5-year EFS: 96.9% vs. 78.3%, *P* = 0.022) in patients aged ≤40 years (Table 4). We further investigated the effect of two chemotherapy regimens on the survival of patients with positive LNs, and they did not show significant improvement in DDFS, EFS, and DDFS in the dose-dense group compared with the standard-interval group (Table 4).

### Left ventricular ejection fraction values at baseline and post-neoadjuvant chemotherapy

Based on the available data from 352 cases, the mean left ventricular ejection fraction (LVEF) values were 68.1% and 67.1% for the dose-dense group and 68.6% and 66.8% for the standard-interval group at baseline (*P* = 0.301) and post-NAC (*P* = 0.624), respectively. There were no significant differences in LVEF declines between the groups (*P* = 0.337).

## Discussion

Dose-dense chemotherapy is one of the standard regimens for patients with high-risk breast cancer^16,17^. With the introduction of anti-HER2 targeted therapy, it remains unclear whether dose-dense chemotherapy still leads to superior survival compared with standard-interval chemotherapy. The present study showed that dose-dense chemotherapy significantly improved the pCR rate of LNs in patients with HER2-positive cancer who received trastuzumab, and improved DDFS and EFS among young patients. Dose-dense chemotherapy also improved the pCR rate of breast tumors, although not significantly. This study is currently, to the best of our knowledge, the largest analysis focusing on the survival of patients with HER2-positive breast cancer receiving dose-dense chemotherapy with trastuzumab. This is also the first study to explore the effect of neoadjuvant dose-dense chemotherapy compared with that of standard-interval chemotherapy in patients with HER2-positive breast cancer receiving trastuzumab.

Previous prospective studies have not compared dose-dense chemotherapy with standard-interval chemotherapy for patients with HER2-positive breast cancer exposed to trastuzumab^7,9,10,15^. The PANTHER trial was a prospective, randomized, phase 3 study focusing on the difference in survival between the dose-dense chemotherapy group and the standard-interval chemotherapy group^8^. The PANTHER trial also recently published a retrospective analysis focusing on HER2-positive breast cancer^15^. With a median follow-up of 5.3 years, 330 patients underwent trastuzumab treatment, of which 29 patients did not receive the complete administration of trastuzumab treatment. The absolute benefit of breast cancer relapse-free survival (BCRFS) in the dose-dense chemotherapy group was 3.8% compared with that of the standard-interval chemotherapy group, but the difference was not significant (HR = 0.68, 95% CI: 0.37–1.27; *P* = 0.231). There was a trend towards improved BCRFS even though trastuzumab was administered. Some of the patients in the PANTHER trial began administration of trastuzumab after completion of chemotherapy, similar to patients in the GIM2 trial^14^. However, some studies have suggested that the concurrent administration of trastuzumab combined with chemotherapy could result in superior survival^18,19^. In addition, the chemotherapy doses in the dose-dense group were adjusted, rather than uniform, according to the hematologic toxic and specific nonhematologic toxic effects on patients, whereas chemotherapy doses were not modified in the control group unless severe toxic effects were observed. This may indicate that no significant differences in survival between the experimental group and the control group were observed. The treatments in the present study were different from those used in the above trial. In our study, chemotherapy regimens were administered in standard doses. This is more likely to reflect the actual differences in survival outcomes between the dose-dense and standard treatment groups.

The GIM2 trial was a prospective, phase 3 study in which patients with LN metastasis were randomized to receive four cycles of (fluorouracil) epirubicin/cyclophosphamide followed by four cycles of paclitaxel delivered either every 2 (dose-dense) or 3 (standard-interval) weeks. In the exploratory analysis from the GIM2 trial, 132 patients with HER2-positive breast cancer were administered with chemotherapy followed by trastuzumab^14^. With a median follow-up of 8.1 years, the comparisons of survival (7-year DFS: 68.7% vs. 72.3%, HR = 0.99, and OS: 84.9% vs. 86.1%; HR = 0.95) between the dose-dense and standard groups showed no significant difference. Compared with our study and the PANTHER study, the number of patients treated with trastuzumab in the retrospective cohort from the GIM2 trial was the lowest, and it was difficult to assess the differences between the dose-dense and standard-interval groups. The introduction of trastuzumab to HER2-positive breast cancer treatment has reduced the risk of recurrence by nearly 50%^11,13,20^. This effect may attenuate the effect of chemotherapy on survival, and more cases will be needed to observe the effect of dose-dense chemotherapy on survival.

After adjusting for related factors, the multivariate analyses in this study showed that LN status and age at diagnosis influenced survival. Further subgroup analyses showed that the application of dose-dense chemotherapy to younger patients can significantly improve survival outcomes. Similar results have been obtained in previous studies^3,7,20^. Patients with high-risk breast cancer who received dose-dense chemotherapy had better survival than did those who received standard-interval chemotherapy^21^. Previous studies also suggested that patients aged ≤40 years with breast cancer seem to have a higher risk of recurrence and poor prognosis than do older patients^22,23^. Thus, 40 years was the age cut-off in this study, and 18.6% of patients were under the age of 40 years. In contrast, some studies in which more low-risk patients (GONO-MIG1) were enrolled, or in which the dose of drugs was lower than that of the control group, found no beneficial effect of dose-dense chemotherapy on survival^5,9^. Thus, dose-dense chemotherapy may bring about more significant survival benefits in young patients with HER-2-positive breast cancer treated with trastuzumab.

Our study found that the LN pCR rate in the dose-dense group was significantly higher than that in the standard-interval group. The breast tumor pCR rate increased by 9.2% when exposed to dose-dense chemotherapy, with a borderline significant difference compared with patients exposed to standard-interval chemotherapy (*P* = 0.052). Untch et al. compared the efficacy of neoadjuvant dose-dense chemotherapy and standard chemotherapy and found that the pCR rate (ypT0/ypTis) in the dose-dense chemotherapy group was significantly higher than that in the standard chemotherapy group (18% vs. 10%, *P* = 0.008)^24^. Even with a strictly defined pCR rate (ypT0, ypN0), the dose-dense chemotherapy group still showed a significant advantage (12% vs. 6%, *P* = 0.011). Some studies believe that pCR in the primary tumor or LN in HER2-positive breast cancer indicates improved survival^25–28^. In our study, with the use of neoadjuvant therapy and trastuzumab, the pCR rate of LNs was higher than that in previous studies^29,30^, suggesting that dose-dense chemotherapy may be more beneficial for LN-positive patients with HER2-positive breast cancer. This effect has some advantages: the previous study found that a higher pCR rate could result in fewer positive SLNB after NAC in patients with clinical negative LN^31^; for the patients with positive LN at initial diagnosis, the improving pCR rate of LNs may provide patients with an opportunity to be exempted from axillary LN dissection after NAC.

Anti-HER2 therapy for HER2-positive breast cancer has gradually developed from single blockade to dual blockade, and intensive targeted therapy after NAC^19,32^. However, the effect of dose-dense chemotherapy with dual HER2 blockade on the survival of patients with HER2-positive breast cancer remains unclear. The TRAIN-2 study suggested that dual HER2 blockade combined with anthracycline-free chemotherapy can achieve a pCR rate similar to that achieved with anthracycline-based chemotherapy^33^. However, the anthracycline-based chemotherapy in the TRAIN-2 study was a 3-week regimen, and the nine cycles of paclitaxel (80 mg/m² on days 1 and 8, every 3 weeks) and carboplatin were not routinely administered in clinical practice, thus the difference between dose-dense and standard-interval chemotherapy delivering dual-targeted therapy could not be confirmed. The present study showed that dose-dense chemotherapy significantly improved the pCR rate of LNs in patients with HER2-positive breast cancer who received trastuzumab; however, it did not achieve significant improvement in the pCR rate of breast tumors. The more patients have the pCR post-NAC, the fewer patients will need TDM1 administration during adjuvant therapy.

Our study had some limitations. First, this was a retrospective study with unknown or unbalanced baseline characteristics. Second, the effect of the combination of trastuzumab with dose-dense chemotherapy on the risk of cardiotoxicity during adjuvant therapy remains unknown. However, in the PANTHER study, no significant differences were noted between the dose-dense group and the standard-interval group in terms of mean LVEF values when trastuzumab was introduced^15^. Little serious adverse events were noted. Third, the sample size of the present study was small, and the follow-up duration was short, hence, we could not observe longer-term events. Therefore, the analysis may not have enough power to predict BCSS in the entire cohort. In the neoadjuvant therapy setting, some pathologic information, such as the pathologic size of the primary tumor, the extent of lymphovascular invasion, and the initial number of involved LNs, could not be accurately obtained after NAC.

In summary, patients with HER2-positive breast cancer who received NAC in combination with trastuzumab achieved a higher LN pCR rate with dose-dense chemotherapy than they did with the standard-interval regimen. Among young patients (aged ≤40 years), neoadjuvant treatment with dose-dense chemotherapy resulted in significantly better survival rates than that in treatment standard-interval chemotherapy. A study with a longer follow-up and larger sample size than used in this study is required to verify the above findings.

## Methods

### Study design and eligibility criteria

Patient data between 2012 and 2016 were retrospectively obtained from the database of Peking University Cancer Hospital. Patients were included based on the following criteria: operable invasive primary breast cancer confirmed by histology, aged 18–70 years, HER-2 positive, exposure to an anthracycline-based regimen followed by paclitaxel with trastuzumab as NAC, duration of trastuzumab administration of 1 year, and completed surgery. Patients were excluded based on the following criteria: M1 stage, breast carcinoma in situ only, synchronous bilateral breast cancer, no NAC or trastuzumab treatment, and a history of malignant tumors. Clinicopathologic data included the tumor size, histological grade, hormone receptor status, LN status, surgical methods, and pathologic remission status. The node-positive disease was confirmed by ultrasound-guided LN biopsy or SLNB before the initiation of NAC. SLNB was performed in patients who initially presented with clinically negative nodes (cN0). Node-negative status was confirmed by a negative SLNB. The study was in accordance with the ethical standards of Peking University Cancer Hospital.

HER2-positive tumors were defined by a finding of HER2 (3+), determined using immunohistochemistry or gene amplification and assessed using fluorescence in situ hybridization. The definition of total pCR was ypT0/is and ypN0. ypT0 was no invasive or noninvasive residual cancer cells in the breast, ypT0/is was no invasive residual cancer cells in the breast, and ypN0 was no residual cancer cells in the LNs.

### Treatment regimens

The regimen of dose-dense chemotherapy was as follows: four cycles of intravenous epirubicin and cyclophosphamide (EC, epirubicin at 100 mg/m^2^ + cyclophosphamide at 600 mg/m^2^ on day 1) every 2 weeks followed by 12 weeks of intravenous paclitaxel at 80 mg/m^2^ on day 1, every 1 week (ddEC-P). Patients in the dose-dense group received prophylactic granulocyte colony-stimulating factor (G-CSF): G-CSF 300 μg subcutaneously on days 4, 6, 8, and 10, or pegfilgrastim 6 mg subcutaneously on day 2 (CSPC Baike [Shandong] Biopharmaceutical Co., Ltd., China). In the standard-interval EC-P group, EC was administered with the same drugs and doses as the dose-dense group every 3 weeks without prophylactic G-CSF. Paclitaxel was administered with the same schedule as the dose-dense group. Trastuzumab was delivered for four 3-weekly cycles along with paclitaxel during neoadjuvant therapy, and was continued alone after surgery to complete 1 year of anti-HER2 treatment. All patients completed 1 year of trastuzumab.

Surgery was mandatory after the completion of NAC without adjuvant chemotherapy in all patients. Primary breast tumors were treated with breast-conserving surgery or mastectomy. For patients with LN metastasis, axillary LN dissection (ALND) was required. ALND could be avoided in patients with a negative SLNB.

Radiotherapy was indispensable for patients who underwent breast-conserving surgery. For patients who underwent a mastectomy, radiotherapy was selected according to the institutional guidelines. Premenopausal patients with hormone-receptor-positive tumors received either tamoxifen 20 mg/d or an aromatase inhibitor plus ovarian function suppression for at least 5 years starting after surgery, and postmenopausal patients received aromatase inhibitor for 5 years starting after surgery.

### Follow-up and outcomes

Duration of follow-up was calculated from the date of neoadjuvant treatment to the date of breast cancer-specific death or last follow-up (censored). The endpoints of this study were BCSS, DDFS, and EFS. BCSS was defined as the time to breast cancer-related death. DDFS was measured from neoadjuvant treatment until distant metastases were observed. EFS events included local recurrence, regional recurrence, distant metastasis, and breast cancer-specific death. Loss to follow-up was defined as no review after the completion of trastuzumab treatment.

### Statistical analyses

Statistical analyses were performed using SPSS 22.0 software (IBM, Armonk, NY, USA). Patient’s baseline characteristics were calculated using frequency tables to show the number and percentage of patients with a particular variable. Patients were divided into two subgroups according to whether they received dose-dense chemotherapy: the dose-dense group and the standard-interval group. Frequency association analyses were performed using the chi-square test or Fisher’s exact method. Survival rates and curves were computed using the Kaplan−Meier method. Differences in survival outcomes between the two groups were determined using the log-rank test. In the multivariate analyses, the Cox proportional hazards model was used to determine the association between related prognosis factors and survival, and to analyze the effect of dose-dense chemotherapy on DDFS, EFS, and BCSS after adjusting for age at diagnosis, tumor size, histological grade, hormone receptor status, axillary LN status, and surgical methods. All statistical tests were two-sided, and a *P-*value < 0.05 was considered statistically significant.

### Reporting summary

Further information on research design is available in the Nature Research Reporting Summary linked to this article.

## Supplementary information

Reporting Summary

## Data Availability

The data generated and analyzed during this study are described in the following data record: 10.6084/m9.figshare.14483553^34^. All data are contained in the file ‘NPJBCANCER-01078_data.xlsx’. This file is not openly available in order to protect privacy. However, the file may be available upon request. Data requests should be made to Dr. Tao Ouyang.

## Code availability

The codes developed during this study are available upon reasonable request.

Analyses were performed using SPSS 22.0 software (IBM, Armonk, NY, USA).
